# In this month’s Bulletin

**DOI:** 10.2471/BLT.20.000420

**Published:** 2020-04-01

This month’s theme issue is on the ethics of applying artificial intelligence to the health sector. In the editorial section, Kenneth Goodman et al. (230) introduce the World Health Organization’s expert group on artificial intelligence in health and Bernardo Mariano (231) describes its global strategy on digital health. Flavia Bustreo and Marcel Tanner (232) imagine inclusive provision of health services in a digital age.  

In the news section, Gary Humphreys (235–236) reports on health sector security risks and regulatory requirements for digital systems. He interviews Tze-Yun Leong (237–238) about the engineering skills needed to develop artificial intelligence capable of dealing with real-life problems. 

## Canada

### Increasing inequities?

Maxwell J Smith et al. (290–292) consider the digital divide, algorithmic biases, competing values and opaque decision-making. 

## Canada, Côte d’Ivoire, Ghana, Finland

### Mobile phone microscopes

Alon Vaisman et al. (288–289) review the ethical quandries of diagnostic imaging for neglected tropical diseases. 

## China, Singapore, United States of America

### Big data analytics in health insurance

Calvin W L Ho et al. (263–269) describe features of a robust ethical and regulatory environment. 

## India

### Individual health and identity

Vijayaprasad Gopichandran et al. (277–281) inspect the challenges of using demographic and biometric data to allocate services.

## Norway

### Towards trustworthy artificial intelligence

Kristine Bærøe et al. (257–187) analyse European Commission guidelines.

## United Kingdom of Great Britain and Northern Ireland

### Population health research 

Gabrielle Samuel & Gemma Derrick (239–244) propose a process for ethics governance. 

### Retaining empathy

Angeliki Kerasidou (245–250) explores trade-offs in automated provision of care.

### Avoiding deaths from sepsis

Ibrahim Habli et al. (251–256) consider the moral accountability of clinical decision-making systems.

### Moral crumple zones

Amy K Paul & Merrick Schaefer (282–284) suggest safeguards for national deployment of digital innovations.  

### Vast linkable data

Claire Louise Thompson & Heather May Morgan (293–295) detail a code-of-conduct for artificial intelligence use in the national health service.

## United States of America 

### Handing over psychiatric research

Nicholas C Jacobson et al. (270–276) explore ethical dilemmas posed by machine learning. 

### Chatbot counselling

David D Luxton (285–287) takes a cautious look at conversational agents.

